# The Importance of an Early and Accurate MEN1 Diagnosis

**DOI:** 10.3389/fendo.2018.00533

**Published:** 2018-09-11

**Authors:** Joanne M. de Laat, Rachel S. van Leeuwaarde, Gerlof D. Valk

**Affiliations:** Department of Endocrine Oncology, University Medical Center Utrecht, Utrecht, Netherlands

**Keywords:** MEN1, diagnosis, genetic testing, epidemiology, delayed diagnosis

## Abstract

Multiple Endocrine Neoplasia type 1 (MEN1) is a rare autosomal dominant inherited condition, causing significant morbidity, and a reduction of life expectancy. A timely and accurate diagnosis of MEN1 is paramount to improve disease outcomes. This enables early identification of tumor manifestations allowing timely treatment for reducing morbidity and improving survival. Current management of MEN1 poses two challenges regarding the MEN1 diagnosis: diagnostic delay and the issue of phenocopies. A delay in diagnosis can be caused by a delay in identifying the index case, and by a delay in identifying affected family members of an index case. At present, lag time between diagnosis of MEN1 in index cases and genetic testing of family members was estimated to be 3.5 years. A subsequent delay in diagnosing affected family members was demonstrated to cause potential harm. Non-index cases have been found to develop clinically relevant tumor manifestations during the lag times. Centralized care, monitoring of patients outcomes on a national level and thereby improving awareness of physicians treating MEN1 patients, will contribute to improved care. The second challenge relates to “phenocopies.” Phenocopies refers to the 5–25% of clinically diagnosed patients with MEN1in whom no mutation can be found. Up to now, the clinical diagnosis of MEN1 is defined as the simultaneous presence of at least two of the three characteristic tumors (pituitary, parathyroids, or pancreatic islets). These clinically diagnosed patients undergo intensive follow up. Recent insights, however, challenge the validity of this clinical criterion. The most common mutation-negative MEN1 phenotype is the combination of primary hyperparathyroidism and a pituitary adenoma. This phenotype might also be caused by mutations in the *CDKN1B* gene, causing the recently described MEN4 syndrome. Moreover, primary hyperparathyroidism and pituitary adenoma are relatively common in the general population. Limiting follow-up in patients with a sporadic co-occurrence of pHPT and PIT could reduce exposure to radiation from imaging, healthcare costs and anxiety.

## Introduction

Multiple Endocrine Neoplasia type 1 (MEN1) is a rare autosomal dominant inherited condition, with a prevalence estimated around 1–10/100 000 (1). MEN1 causes significant morbidity and reduction of life expectancy in those affected (2–4). Penetrance of MEN1 is high, over 90% of individuals carrying a *MEN1* mutation will be affected (2, 3, 5). A timely and accurate diagnosis of index cases and their family members is key to management of MEN1. There is mounting evidence that intensive follow-up of MEN1 patients and screening for tumor manifestations reduces morbidity and improves survival (3, 6). Conversely, a delay in MEN1 diagnosis has been found to cause potential harm (7). In a recent study the lag time between diagnosis of MEN1 in index cases and genetic testing of family members was 3.5 years. Before or during this lag time several familial cases already had developed metastatic neuroendocrine tumor manifestations (7).

The diagnostic approach to patients in whom MEN1 is suspected has rapidly evolved since the discovery of the *MEN1* gene in 1997 located at 11q13 (1, 8). Nowadays, genetic testing has a well-established role in confirming diagnosis of MEN1, identifying family members of index patients with *MEN1* mutation who are at risk to develop tumor manifestation, and reassuring family members without a mutation.

Nevertheless, new diagnostic challenges have also emerged. In 5–25% of patients with a clinical diagnosis of MEN1 no mutation can be found, a phenomenon often referred to as “phenocopies” (9, 10). There is increasing debate if such patients are correctly diagnosed as having MEN1 since some of these patients might have a sporadic coincidence of two neuroendocrine tumors (3). However, there are also mutations in other genes that can cause a MEN-1 like phenotype. Mutations in the cyclin-dependent kinase inhibitor (*CDNK1B*), have been found to cause a syndrome of parathyroid and anterior pituitary tumors (11). Patients with a *CDNK1B* gene mutation have a clinical course different from patients with *MEN1* mutations, and have a lower risk to develop the pancreatic neuro-endocrine tumors (pNET). For this reason the syndrome is now referred to as MEN4.

Another diagnostic challenge is to reduce a diagnostic delay of MEN1. A delay in diagnosis can be caused by a delay in identifying the index case, and by a delay in identifying affected family members after diagnosing an index case. Identifying index cases raises the question “who and when to screen for a *MEN1* mutation?.” Incidence of some manifestations related to MEN1 is high in the general population. The incidence of primary hyperparathyroidism (pHPT) for example is estimated as high as 34 to 120 per 100,000 person-years among women, and from 13 to 36 among men (12). Current guidelines advice genetic screening for MEN1 in patients with pHPT if diagnosed at an age below the age of 30 years (or below the age of 40 years in multigland parathyroid disease). Concern has been raised that such policy might results in a significant delay in diagnosis of the index case (13–15). Reducing lag times in identification of *MEN1* mutation carriers among family members of MEN1 patients requires awareness among treating physicians and organized structured program (7, 16, 17).

In this review we will discuss implications of recent epidemiological insights on clinical diagnostic criteria for MEN1 and the “phenocopies” phenomenon. Furthermore we will assess the impact of delay in diagnosis and discuss medical and organizational advances that can help reducing lag times in both index cases and their family members (Table 1).

**Table 1:** Review highlights.

- Delay in diagnosis of MEN1 can result in significant harm to patients and their family members
- Family members of MEN1 patients should be offered genetic testing at the earliest account
- Criteria for *MEN1* testing have been outlined in the clinical practice guidelines. Delay of diagnosis in index cases might further be reduced by considering family history, and assessing the individual risk of a *MEN1* mutation using a prediction model.
- In 10–25% of patients with a MEN1 phenotype no *MEN1* mutation can be found
- In a patient with a pHPT and PIT phenotype and no *MEN1* mutation, other syndromes like FIPA and MEN4 should be considered as well as a sporadic co-occurrence of two neuroendocrine tumors.
- Limited follow-up can be considered in patients with MEN1-phenotype based on FIPA, MEN4, or sporadic co-occurrence of pHPT and PIT; limited follow-up potentially reduces exposure to radiation from imaging, costs, and anxiety.

## Clinical diagnosis of MEN1

In the early 20th century simultaneous occurrence of three tumors characteristic for MEN1 (pituitary, parathyroids, and pancreatic islets) had been reported as a pathological rarity (18, 19). In 1953, Underdahl et al. described a clinical diagnosis of MEN1 based on a case series of eight patients with a syndrome of multiple endocrine adenomas including a literature study comprising another fourteen cases (20). In addition to the tumors of the pituitary, parathyroids and pancreatic islets, three of his eight patients presented with peptic ulcers and one patient had an adrenal adenoma. Wermer et al. found that the syndrome was autosomal dominantly inherited, describing the cases of four sisters and their father with similar tumors (21).

Up to now, the simultaneous presence of at least two of the three characteristic tumors (pituitary, parathyroids or pancreatic islets) is still considered pathognomic for MEN1 (10). The current clinical practice guidelines describes three criteria for diagnosis of MEN1:(10)

- The genetic criterion: presence of a known *MEN1* mutation, irrespective of clinical or biomechanical manifestation.
- The familial criterion: occurrence of at least one MEN1 associated tumor and a first-degree relative with MEN1.
- The clinical criterion: defined by the occurrence of at least two out of three characteristic tumors (pituitary, parathyroid, or pancreatic islets).

The clinical criterion, however, has now become subject to debate. Genetical testing in MEN1 patients meeting the clinical criterion is negative in up to 25% of cases (10). Negative mutation analysis is predominantly found in patients without a family history of MEN1 (14, 15, 22, 23). Till now, these patients were considered and treated as new index cases of MEN1. With the introduction of new highly sensitive genetic tests such as multiplex ligation-dependent probe amplification (MLPA), incidence of negative genetic testing in patients clinically diagnosed with MEN1 indeed declined, but still is around 10% (9, 24, 25). It is questionable whether all patients meeting the clinical criterion and a negative mutation analysis have MEN1 since clinical observations show that mutation negative patients often have a more favorable clinical course (3, 14, 22).

Negative mutation testing is most prevalent in the clinical MEN1 phenotype comprising a combination of tumors of the parathyroids and pituitary gland. In a study from Hai et al. reporting on 20 clinically diagnosed index cases of MEN1, frequency of *MEN1* mutation was only 11% in nine patients presenting with a combination of tumors of parathyroids and pituitary glands vs. a 63% frequency in eleven patients with other tumor combinations (22). These results were confirmed in a study by Klein et al. (26). In twenty index cases undergoing mutational analysis in this study, none of the ten patients presenting with tumors of parathyroids and pituitary glands tested positive for *MEN1* mutation, vs. 60% of patients in whom phenotype included a pancreatic tumor. Interestingly, frequency of *MEN1* mutation was also only 25% in 32 pedigrees of familial occurrence of tumors of parathyroids and pituitary gland (26).

Alternative explanations for negative testing in patients with clinical diagnosed MEN1, or “phenocopies,” should be considered. There are either other syndromes that can cause a MEN1-like phenotype or sporadic co-incidence of two neuro-endocrine tumors.

### Other syndromes that can cause a MEN1-like phenotype

Two syndromes that should be considered in mutation-negative MEN1 patients that are of particular interest are MEN4 and Familial Isolated Pituitary Adenomas (FIPA). MEN4 is a syndrome of parathyroid and anterior pituitary tumors that is caused by mutations in the *CDKN1B* gene, which encodes the cyclin-dependent kinase inhibitor p27^kip1^ (11). Mutations in this gene were first found to cause a syndrome of neuroendocrine tumors in rat studies, in patterns that could overlap both MEN1 and MEN2 (27). Pellegata et al. subsequently reported a *CDKN1B* gene mutation in a patient presenting with pituitary and parathyroid tumors, in whom analysis of his pedigree revealed MEN1-like phenotypes in multiple generations (28).

Several other reports of *CDKN1B* mutations have been made (3, 29–34). Most reported on cases of parathyroid and anterior pituitary tumors, besides one case of a pituitary tumor in combination with a well differentiated pancreatic neoplasm (32). Agarwal et al. screened 196 patients with MEN1 phenotype who scored negative for *MEN1* mutation analysis for MEN4 and mutation in other cyclin-dependent kinase inhibitors (33). In their analyses, seven potentially pathological mutation in cyclin-dependent kinase inhibitors, 3 of which in p27 related to MEN4 are described. Compared to MEN1 patients, patients with MEN4 develop tumors at a relatively late age with a mean age for developing pHPT at 56 years (29–31, 33, 34). Little is known about long-term follow-up of MEN4 because of the small number of patients reported and limited follow-up.

FIPA is a syndrome of familial occurring isolated pituitary adenomas caused by a mutation in the *AIP* gene, most commonly resulting in prolactinomas and GH- secreting pituitary tumors (PIT) (35, 36). Patients with FIPA develop pituitary tumors at a relative younger age as compared to sporadic cases (36). FIPA syndrome in itself will not result in a complete MEN1- like phenotype. However patients with FIPA need only to develop one sporadic neuroendocrine manifestation (of which pHPT is not uncommon in the normal population) to meet clinical criteria for MEN1. In combination with the familial occurrence of PIT in patients FIPA, one can easily imagine that some patients with FIPA might mistakenly be diagnosed as having MEN1. Georgitsi et al. reported on screening for *AIP* mutation in 490 patients with PIT, 91 of whom were previously screened for *MEN1* (37). Two of 91 patients previously suspected for MEN1 an *AIP* mutation was found. Both patients developed a GH-secreting PIT at young age (16 and 18 years respectively). The report did not mention if these patients had also developed other MEN1 related manifestations.

No specific guidelines for follow-up of patients with FIPA and MEN4 exist. Nonetheless, it seems safe to apply a much more limited screening program to patients with FIPA and MEN4 as compared to patients with true MEN1. For patients with FIPA, Korbonits et al. recommend yearly clinical assessment and pituitary function tests, accompanied by dynamic testing to evaluate for hormone excess or deficiency as needed, and follow-up pituitary MRI (38). In patients with MEN4 the need for thoracic imaging, as performed in patients MEN1 for early diagnosis of bronchopulmonary NET and thymic tumors, might be waived. Screening for pancreatic NET in MEN4 can be debated. Sporadic cases of pancreatic NET in patients with MEN4 have been reported, although the incidence seems much lower than in patients with MEN1 (32).

Syndromes such as MEN4 and FIPA predisposing for neuroendocrine tumors can cause a MEN1-like phenotype and need to be considered in mutation- negative patients. However, these syndromes might explain only a minority of the total of mutation- negative MEN1 phenotypes. Georgitsi et al. screened 106 patients to find one case of MEN4, as compared to Agarwal et al. who found 3 cases in 196 patients (29, 33). In a national cohort study from the Netherlands, de Laat et al. found only one *CDKN1B* mutations and no *AIP* mutations upon additional screening among 30 mutation-negative MEN1 patients (3). Igreja et al. found no *CDKN1B* or *AIP* mutation in 21 patients mutation-negative MEN1 patients (39). Several other papers testing mutation-negative patients for *CDKN1B* mutation, but not AIP, could not demonstrate a pathologic mutation (40, 41).

### Sporadic co-incidence of two neuroendocrine tumors

Another explanation for mutation negative MEN1 phenotype is a sporadic co-incidence of two neuro-endocrine tumors. Such sporadic co-incidence might be much more common than generally perceived. The prevalence of pHPT is relatively high in the normal population, and has been rising over the past decades. In recent years, a prevalence of 233 per 100,000 women and 85 per 100,000 men has been reported (12). With improving imaging techniques, prevalence of incidental PIT is also increasing. The clinical significance of such incidentalomas is still uncertain. The current clinical practice guideline for pituitary incidentalomas advises lifelong radiological follow-up (42). In a healthy volunteer study including 100 subjects pituitary incidentalomas on MRI were prevalent in 10% (43). A systematic review even reported incidental pituitary adenomas in 22.5% of cerebral imaging studies (44). Reflecting these numbers, a co-incidence of pHPT and an incidental pituitary adenoma could occur without the necessity for a tumor predisposition that requires intensive follow-up.

The findings of a long-term follow-up study comparing between 293 mutation-positive and 30 mutation-negative MEN1 patients in the Netherlands indeed showed that the mutation-negative patients have a very mild natural course of disease with a life expectancy that is comparable to that of the general Dutch population (3). As in other studies, a combination of pHPT and PIT was the most prevalent phenotype (77%) among mutation-negative patients. At the time of this study all mutation-negative patients were index cases without a familial history of MEN1. Later a sibling of one of the mutation-negative patients also developed a clinically diagnosed MEN1 (7). None of the 30 mutation-negative patients developed a third MEN1 manifestation during a median of 8 years of follow-up. Also none of the mutation-negative patients died from a MEN1 related cause as opposed to the mutation positive group in whom 60% of mortality was related to MEN1. Finally, median survival in mutation-negative patients was 84.0 years (95% CI: NA) compared to 73.0 years (95% CI: 69.3-76.6) in mutation-positive patients (*P* = 0.013) (3). Both this Dutch cohort and a Japanese cohort showed that mutation-negative patients develop tumor manifestations at a significant later age, supporting the evidence for a milder natural course (3, 22).

It is impossible to exclude that clinically diagnosed mutation-negative MEN1 patients, might harbor a yet unknown mutation to either the *MEN1* gene or any other gene that predisposes for neuroendocrine tumor development. Nevertheless, it seems reasonable that many such patients have a sporadic co-incidence of two tumors and that at least part of the mutation-negative MEN1 patients can be discharged from intensive follow-up. Hereby it is important to recognize that patients with genetically proven MEN1, who are systematically followed-up, have a high fear of disease occurrence which is associated with a lower quality of life (45). In addition, intensive follow-up in subjects without a high risk of tumor occurrence leads to overutilization of health care resources and costs.

A key issue remains how to select patients that can be discharged from follow-up. The first important modulating factor to consider is family history. A diligent pedigree analysis remains one the most important clinical tools to raise suspicion about a yet unknown inheritable disorder.

Because most mutation-negative patients will present with pHPT, it is necassary to consider modulating factors in the clinical presentation of pHPT, i.e., multiglandular vs. single gland disease. Hyperparathyroidism in true MEN1 patients typically present as “asymmetrical hyperplasia” affecting multiple glands, for this reason a subtotal hyperparathyroidectomy is considered the optimal treatment for pHPT in MEN1 (4, 46, 47). Although this has been criticized in a recent retrospective cohort 8/24 patient with pHPT was treated by unilateral clearance with a 87.5% success rate (48). The study unfortunately did not report on mutation status of these patients or further manifestations.

In an additional analysis of the Dutch cohort of mutation-negative patients the pathology and imaging results of 28/30 (93.3%) mutation-negative patients presenting with pHPT were reviewed (3). In 22 of the 28 mutation negative patients with pHPT an parathyreoidectomy was performed. The pathology showed a uniglandular adenoma in all cases. Six mutation negative patients with pHPT were not operated, however in 3 out of these 6 patients imaging results suggested uniglandular disease (ultrasound or Tc99m-sestamibi parathyroid scintigraphy). In conclusion, mutation-negative patients with pHPT not only present with a significantly better clinical course but also predominantly presented with a uniglandular pHPT. An uniglandular pHPT therefore seems to increase the likelihood of co-incidence of two sporadic neuroendocrine tumors instead of true MEN1.

Summarizing these epidemiological data, we propose an approach to clinically diagnosed MEN1 patients based upon co-occurrence of pHPT and PIT without a *MEN1* mutation. A MEN4 or FIPA diagnosis should be considered in these patients. If such mutation is found, follow-up as appropriate for these conditions should be provided. If no mutation is found in these genes, the familial history and the presentation of pHPT should be carefully reviewed. Patients without family history of neuroendocrine tumors or other MEN1 manifestations and “only” uniglandular parathyroid disease might safely be discharged from further intensive follow-up. Limiting follow-up in patients with evidence for MEN4, FIPA, and sporadic co-occurrence of pHPT and PIT could reduce exposure to radiation from imaging, healthcare costs and anxiety.

## Delay in diagnosis of MEN1

There is broad consensus on the need for timely recognition of MEN1. First degree family members of MEN1 patients should be offered genetic testing at the most early account (10). Pediatric cases of MEN1 manifestations have been described as early as 5 years, and several cohort studies indicate that close follow-up and treatment improves survival in *MEN1* gene mutation carriers (2–4, 10). For this reason, the clinical practice guidelines also recommend start of the follow-up program in early childhood. Pediatric manifestations of MEN1, however, are relatively rare, and physicians should weigh the potential benefits against potential harm from radiation by imaging studies, quality of life and costs in the very young patient. Because no apparent genotype/ phenotype relation have described for MEN1 it is difficult to recommend about individualization of MEN1 follow-up.

Delays in the diagnosis of MEN1 can occur by a delay in identifying an index case, or by lag time between diagnosing the index case and testing of family members. Because MEN1 is a rare disease, delays in identification can possibly occur by a lack of awareness about the disease and the indications for genetic screening by treating physicians. A recent review of the Italian MEN1 registry revealed that the average age of first MEN1 manifestation was 41.6 years, while the average age of MEN1 diagnosis was 55.1 years, suggesting a significant potential to improve the time between first manifestation and diagnosis of MEN1 (16).

### Delay in diagnosis of index case

According to the current practice guidelines genetic mutation analysis should be offered to all patients meeting the familial or clinical criteria for diagnosis of MEN1, and patients presenting with a MEN1 related tumor. Suspicion for MEN1 is described as: parathyroid adenoma below the age of 30 years (or multiglandular parathyroid disease at any age); gastrinoma or multiple pancreatic NET at any age or individuals who have two or more MEN1-associated tumors that are not part of the classical triad of parathyroid, pancreatic islet and anterior pituitary tumors (e.g., parathyroid tumor plus adrenal tumor) (10).

Indications for genetic testing in patients presenting with pHPT have been subject to debate and are slowly evolving. The incidence of pHPT in the general population is high, and genetic screening laborious and costly. Thus testing for MEN1 in all patients with pHPT is not considered a cost-effective approach. Previous guidelines recommended testing for MEN1 only in patients presenting with pHPT before the age of 30 (49). Lassen et al. reported on a case of a 31 year old woman presenting with multiglandular pHPT who was initially not tested under this previous guidelines. Diagnosis of MEN1 was only established 7 years later when she developed recurrent disease after subtotal parathyroidectomy (13). In the current clinical practice guidelines indication for *MEN1* mutation analysis has therefore been expanded to forty years in case of multiglandular disease.

Concerns over delayed diagnosis in patients presenting with pHPT, however, are still not fully met. Analysis of referrals for genetic testing in both Sweden and the Netherlands revealed that physicians in both countries frequently referred patients that did not meet the criteria for genetic testing according to the clinical practice guidelines (64 and 81% of all patients referred for genetic counseling respectively) (15, 50). Moreover, in both groups *MEN1* mutations were found among the patients who were tested but did not meet the clinical practice guideline criteria.

Concerns over delayed diagnosis have also been raised in index patients presenting with a pancreatic NET (51). The ENETS consensus guidelines for the management of pancreatic NET's recommend testing for MEN1 in patients presenting with insulinoma before the age of 20, in addition to testing of patients with multiple pancreatic NET's at any age (51). Two recent studies revealed that in 10–12% of MEN1 patients younger than 21 years insulinomas were the first presentation of MEN1(52, 53). This is in line with studies confirming that insulinomas in particular present at young age in patients with MEN1, as opposed to gastrinomas (54, 55). Type 2 well differentiated gastric neuoendocrine neoplasms have been associated with gastrinomas and MEN1 (56). Since nonfunctioning pancreatic NET's also occur from a young age, MEN1 testing should perhaps not only be limited to patients with insulinomas before the age of 20, but recommended for all patients with pancreatic NET's before this age.

Only a relative small percentage of bronchial NET are associated with MEN1 (< 5%). The ESMO and ENETS guideline statement on genetic testing for MEN1 in patients with bronchial NET are in agreement with the clinical practice guidelines for MEN1 (10, 57–59). Testing is adviced if the familial history is suggestive of MEN1 or a second MEN1 feature is present, e.g., hyperparathyroidism (57). Several cohorts report thymic NET in MEN1 patients to occur predominantly in men with a mean age around the fifth decade (60–62). Nevertheless, in a large Japanese cohort of MEN1 patients a relative high percentage (36%) of women was found among patients with thymic NET (63). Thus MEN1 should still be considered in female patients presenting with thymic NET.

Several authors have emphasized the role of family history as a prognostic factor (50, 64, 65). Not only a family history positive for MEN1 should be considered a risk factor for the disease, but also a family history positive for pHPT or any other neuro endocrine tumor up to third degree family members is significantly correlated with an increased risk of finding a *MEN1* mutation.

**Figure 1:**
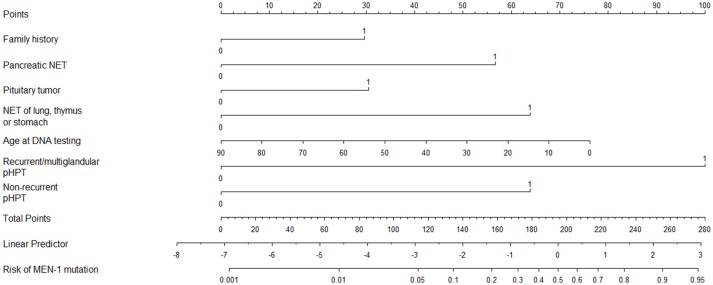
Nomogram for predicting the risk of a MEN1 mutation. NET, neuro- endocrine tumor; pHPT, primary hyperparathyroidism. How to use: Nomogram to calculate the risk of a MEN1 mutation. Draw a vertical line for each variable to the “points” axis at the top. Sum the points for the eight variables and locate this total score on the “total points” axis. Draw a vertical line from this through the bottom two scales to determine the linear predictor and the predicted risk of a MEN1 mutation. Example: a 54-year-old patient (score = 30 points) with the combination of a negative family history. (score = 0 points), a nonrecurrent and nonmultiglandular pHPT (score = 63 points), and a pNET (*n* = 57 points) has a sum score of 150 points, corresponding with a linear predictor of −0.50 and a risk of 38% of having a MEN1 mutation. Example: a 41-year-old patient (score = 42 points) with a positive family history (score = 29 points) and recurrent pHPT (score = 100 points) has a sum score of 171 points, corresponding with a linear predictor of 0.50 and a risk of 63% of having a MEN1 mutation. Originally appeared in: de Laat, J. M., et al. (50).

Based on the Dutch cohort a prediction model was made which was validated in an independent Swedish cohort. This prediction model that allows clinicians in daily clinical practice to estimate the individual risk for a *MEN1* mutation in their patients (50). Based on the risk factors for MEN1 in individual patients, the risk for having a positive MEN1 mutation risk can be calculated which can be used in counseling patients at higher risk for MEN1. Risk factors from this model include: recurrent or multiglandular pHPT; nonrecurrent pHPT; pancreatic and duodenal NET; pituitary tumor; NET of stomach, thymus or bronchus; and positive family history (up to third degree relatives) for any neuroendocrine tumor (Figure 1).

### Delay in diagnosis of MEN1 in family members of patients

In the nationwide Dutch MEN1 study, van Leeuwaarde et al. recently systematically described lag times between diagnosis of a MEN1 index case and testing of family members (7). Delayed genetic testing of family members appeared to be an important cause for avoidable morbidity and even mortality. MEN1 patients are prone for severe morbidity such as osteoporosis caused by Phpt (66, 67). Moreover, higher severity of bone involvement in comparison with sporadic pHPT has been reported (68). Complications due to bone mineral loss and urolithiasis caused by pHPT are early onset and thus have the potential to be progressive and severe (68, 69).

In the Dutch study, 304 MEN1 patients from 58 MEN1 families were included. The median lag time between diagnosis of the index cases and family members was 3.5 years. At the time of the subsequent MEN1 diagnosis in the family members of the index cases, 30 (12.1%) had a duodenopancreatic neuroendocrine tumor, of whom 20% already had metastatic disease. Mean lag time of patients with metastatic disease was 10.9 years, compared to 7.1 years in patients without metastases. Almost 40% of non-index cases had a pHPT at time of diagnosis. A total of five patients had a macroadenoma of whom two had compression of the optic chiasm. Ten non-index cases died because of a MEN1-related cause that might have been developed during the lag time.

This first report on lag time in diagnosis of non-index cases showed alarming outcomes. Reducing the delay in diagnosis of non-index cases requires national efforts to centralize care for MEN1 patients ensuring optimal quality of care. In this study a reduction in lag time from a median of 8 years before 1998 till 0.75 years after 2007 was found (7). During this period MEN1 care was centralized in the Netherlands. Centralized care and monitoring of outcomes in MEN1 patient care through national registries has been well established in a number of European countries as well, including the Group d'etude des Tumeurs Endocrine in France and the Italian network of MEN1 referral centers (16, 61). Centralization and collaboration on a national level in the Netherlands has improved awareness of MEN1 in physicians treating patients with MEN1, which probably has contributed to the reduction of lag times.

In conclusion, a timely MEN1 diagnosis in index cases and their affected family members should be pursued by physicians that treat patients with MEN1. Evidently, not all physicians are aware of the disease and the consequences arising from a delayed diagnosis. Ongoing (inter)national publications and scientific meetings in collaboration with patient advocacy groups will further increase this awareness. In spite of the rarity of the disease, the MEN1 landscape in respect of an accurate MEN1 diagnosis is evolving and still open for debate and improvement. Future cost effectiveness studies could be useful to enhance this discussion and a helpful tool for physicians and policy makers for both clinical and policy wise MEN1 decision-making.

## Author contributions

JdL conception of the work, analysis of available literature, drafting the article, final approval. RvL and GV conception of the work, analysis of available literature, critical revision of the article, final approval.

### Conflict of interest statement

The authors declare that the research was conducted in the absence of any commercial or financial relationships that could be construed as a potential conflict of interest.
